# Bridging-driven condensation by eukaryotic SMC complexes is a conserved feature of genome organization

**DOI:** 10.1093/nar/gkag850

**Published:** 2026-09-03

**Authors:** Jae-Won Jang, Torahiko L Higashi, Minzhe Tang, Changyeop Kim, Yoshimi Kinoshita, Hajin Myeong, Joonyoung Lee, Tomoko Nishiyama, Won-Ki Cho, Frank Uhlmann, Cees Dekker, Je-Kyung Ryu

**Affiliations:** Department of Physics and Astronomy, Seoul National University, Seoul 08826, South Korea; Interdisciplinary Program in Computational Sciences, Seoul National University, Seoul 08826, South Korea; Chromosome Segregation Laboratory, The Francis Crick Institute, London NW1 1AT, United Kingdom; Chromosome Segregation Laboratory, The Francis Crick Institute, London NW1 1AT, United Kingdom; Department of Biological Sciences, Korea Advanced Institute of Science and Technology, Daejeon 34141, South Korea; Division of Biological Sciences, Graduate School of Science, Kyoto University, Sakyo, Kyoto, Japan; Department of Physics and Astronomy, Seoul National University, Seoul 08826, South Korea; Interdisciplinary Program in Genetic Engineering, Seoul 08826, South Korea; Department of Physics and Astronomy, Seoul National University, Seoul 08826, South Korea; Division of Biological Sciences, Graduate School of Science, Kyoto University, Sakyo, Kyoto, Japan; Department of Biological Sciences, Korea Advanced Institute of Science and Technology, Daejeon 34141, South Korea; Chromosome Segregation Laboratory, The Francis Crick Institute, London NW1 1AT, United Kingdom; Department of Bionanoscience, Kavli Institute of Nanoscience Delft, TU Delft, Delft, the Netherlands; Department of Physics and Astronomy, Seoul National University, Seoul 08826, South Korea; Interdisciplinary Program in Computational Sciences, Seoul National University, Seoul 08826, South Korea; Interdisciplinary Program in Genetic Engineering, Seoul 08826, South Korea

## Abstract

The Structural Maintenance of Chromosome (SMC) protein family plays a central role in higher-order genome organization through ATP-dependent DNA loop extrusion by cohesin and condensin and other processes. Whether these activities fully account for the complexity of chromosome architecture remains unknown. Here, we uncover a conserved ATP-independent mechanism of chromatin condensation by SMC complexes, occurring via biomolecular condensation. Using single-molecule fluorescence imaging, we show that a variety of SMCs form dynamic DNA-bound condensates that exhibit key features of biomolecular condensates, including droplet coalescence, fluorescence recovery after photobleaching, and rapid exchange with free SMC complexes. Atomic force microscopy analysis of human cohesin–DNA assemblies reveals DNA-length-dependent clustering, providing evidence for bridging-driven condensation. Analyses of *in vivo* super-resolution imaging and high-throughput chromosome conformation capture (Hi-C) data indicate that these condensates form chromatin-associated clusters with multi-loop structures. Together, our results establish that SMC complexes employ ATP-independent phase condensation as well as ATP-dependent activities to shape genome architecture. This work reveals a broadly conserved principle of chromosomal organization across eukaryotes.

## Graphical Abstract

**Graphical Abstract:**
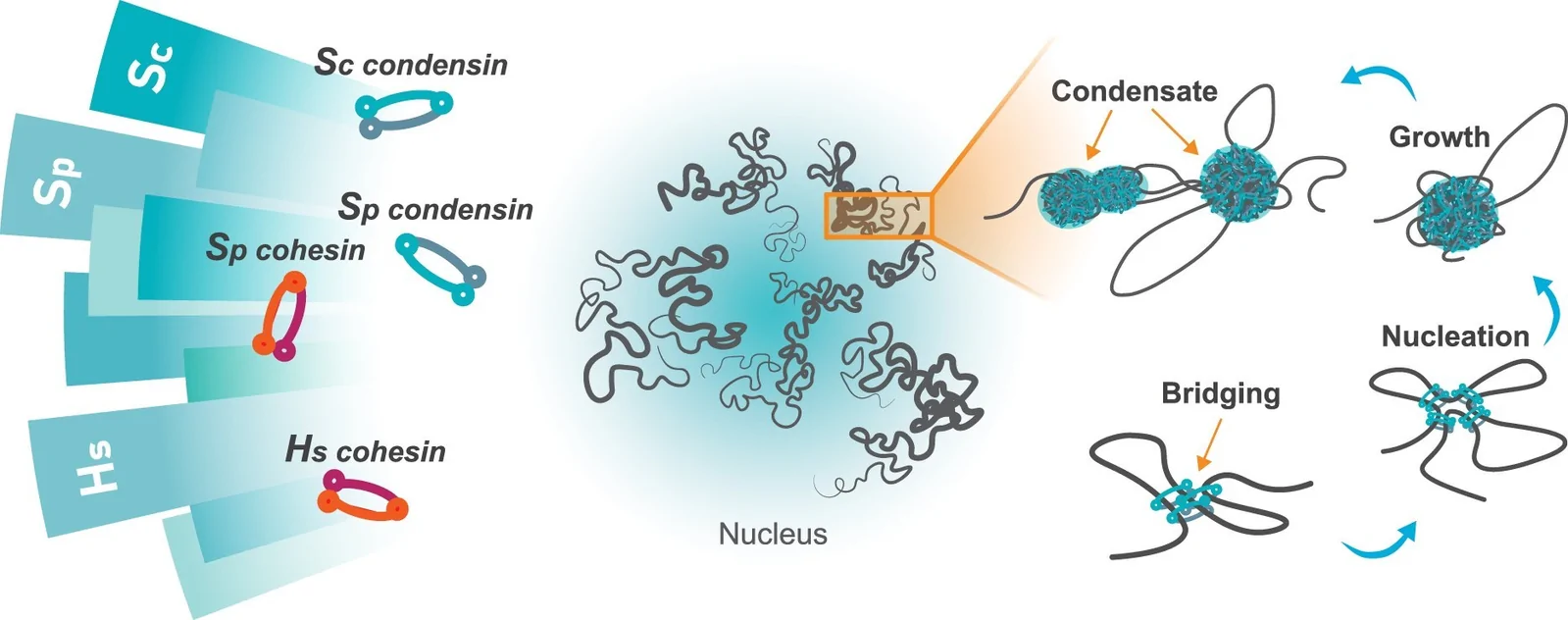

## Introduction

The Structural Maintenance of Chromosome (SMC) protein family plays a fundamental role in genome organization and segregation across all domains of life [1–4]. In eukaryotes, cohesin, condensin, and SMC5/6 complexes mediate large-scale chromatin folding and dynamics, with ATP-dependent DNA loop extrusion and topological DNA embracement emerging as core organizing principles. Single-molecule imaging studies have directly visualized loop extrusion by SMC complexes, offering mechanistic insight into how cohesin shapes interphase chromatin and how condensin compacts mitotic chromosomes into their characteristic architecture [5–13].

While DNA-loop extrusion is widely accepted as a central mechanism of genome folding, it may not solely account for the diversity and complexity of chromosome architecture [14, 15]. Notably, SMC complexes form clusters *in vivo* and exhibit ATP-independent condensation *in vitro* [16–20]. Furthermore, loop extrusion alone cannot explain certain features of mitotic chromosome compaction such as axial shortening and stiffness [21]. These observations suggest the existence of additional, extrusion-independent mechanisms through which SMC complexes contribute to genome organization. Recently, we identified a distinct mechanism termed bridging-induced phase separation (BIPS), in which SMC complexes such as yeast cohesin bridge distant DNA segments to nucleate ATP-independent phase condensation along chromatin [16, 22, 23]. Unlike canonical liquid–liquid phase separation driven by multivalent protein–protein interactions, BIPS arises from multivalent protein–DNA bridging, which may represent a complementary mode of chromatin organization [22–25].

Whether BIPS is a conserved feature of other SMC complexes across diverse species remains unknown. Establishing this is essential, because a mechanism found in only one complex or species could reflect an idiosyncratic biochemical property, whereas conservation across divergent SMC complexes would indicate that bridging-driven condensation is a fundamental principle of genome organization rather than an incidental feature. In this study, we investigate the phase condensation behaviors of cohesin and condensin complexes from *Saccharomyces cerevisiae* (*Sc*), *Schizosaccharomyces pombe* (*Sp*), and *Homo sapiens* (*Hs*). Our findings reveal that SMC-mediated condensation is conserved across complexes and species, suggesting that SMCs employ dual mechanisms—loop extrusion and BIPS—to organize the genome. The results thus uncover a broadly conserved biophysical principle that integrates enzymatic and self-organizing properties of SMC assemblies in shaping chromosomal architecture.

## Materials and methods

### Protein purification

*Sc* condensin, *Sp* condensin, *Sp* cohesin, and *Hs* cohesin were purified by following the previous protocols [11, 16, 26–29].

### Fluorescent labeling of SMC complexes

To label *Sp* cohesin and condensin, we mixed SNAP-tag cohesin tetramer with SNAP-Surface Alexa Fluor 647 (New England Biolabs) in a 20 mM Tris (pH 7.5), 150 mM NaCl, 0.05% (w/v) Tween 20, 3% (w/v) glycerol, bovine serum albumin (BSA; 0.1 mg/ml), and 1 mM dithiothreitol and incubated the mixtures overnight. Labeled protein was separated from free fluorophore using a Zeba Microspin 40 kDa (Thermo Fisher Scientific), we filtered free fluorophores in 20 mM Tris, 150 mM NaCl, 0.05% Tween 20, and 3% glycerol [16]. For *Sc* condensin labeling, we introduced ybbR acceptor peptide (GTDSLEFIASKLA) at the N-terminus of the Brn1 by replacing residues 13–23 [5]. Condensin complexes containing the ybbR tag were purified from yeast cells and incubated with CoA-ATTO647N in buffer containing 10 mM MgCl_2_ and 1.2 μM Sfp phosphopantetheinyl transferase (P9302, New England Biolabs) at 6°C for 16 h in the dark. CoA-ATTO647N was synthesized by incubating ATTO647N-maleimide (AD 647N-41, ATTO-TEC) with 45.5 nmol coenzyme A sodium hydrate (C3144, Sigma) in 0.1 ml of a degassed DMSO:100 mM sodium phosphate buffer (1:1, pH 7.0), followed by addition of 100 nmol of Tris(2-carboxyethyl)phosphine (646547, Sigma) and quenching with dithiothreitol. Free dye and Sfp synthase were eliminated by size exclusion chromatography. For *Hs* cohesin labeling, STAG1-cohesin and HeLA cohesin with Janelia Fluor 646 (JF646), JF646-HaloTag Ligand was synthesized by reacting 1 mg JF646 SE (6148, Tocoris) with 50 μl of 120 mM HaloTag Amine (O2) Ligand (P6711, Promega) in 200 μl DMF, followed by addition of 10 μl diisopropylethylamine (Sigma) [7]. The reaction was incubated for 16 h at room temperature in the dark. Then diluted and purified by five rounds of reverse phase HPLC using Ultimate 3000 (Thermo Fisher Scientific). Fractions were pooled, lyophilized, and resuspended in DMSO. Then purified HaloTag-fused cohesin was incubated on FLAG-M2 agarose beads with excess JF646-HaloTag Ligand in purification buffer for 15 min at room temperature in the dark. Beads were washed, and labeled protein was eluted.

### Preparation of biotin-labeled λ-DNA

We followed the protocol described in the previous literature [16].

### Single-molecule imaging

Microfluidic flow chambers for the single-molecule fluorescence imaging were constructed following an established protocol [30], with chamber dimensions of 3 mm by 15 mm by 100 μm. A quartz slide, drilled with 12 holes, was cleaned with piranha solution. Both the quartz slide and cover slips were PEGylated by dissolving a 1:100 ratio of biotin-PEG and PEG in a sodium bicarbonate solution (0.1 M NaHCO₃, pH 8.5). After rinsing and drying the PEGylated surfaces, chambers were assembled using double-sided tape to create six channels, with epoxy glue sealing the edges. Polytetrafluoroethylene tubing was connected to one side of the chamber’s drilled holes, and a pipette tip was added to form a reservoir.

**Figure 1.**
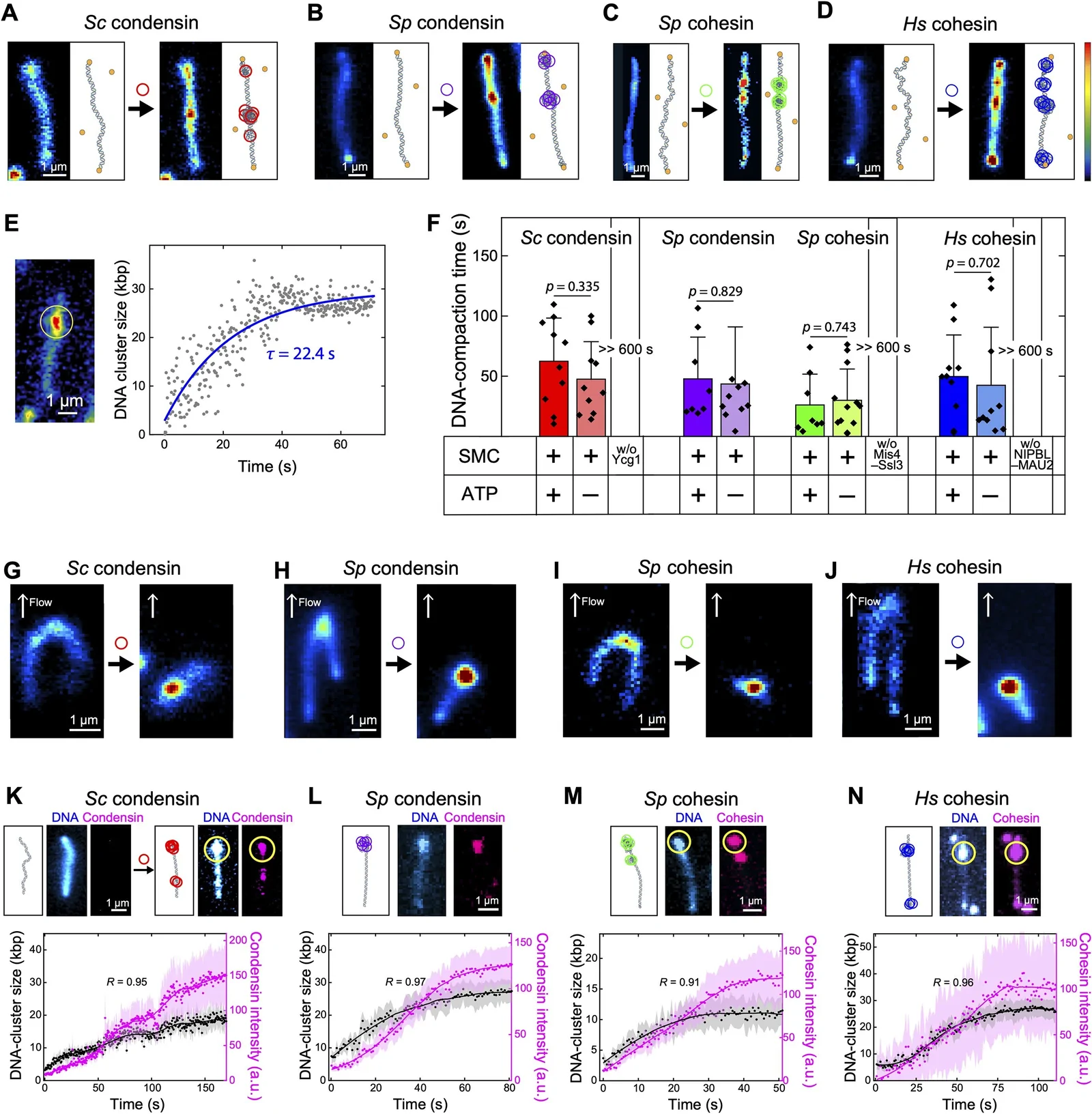
Collective DNA/SMC cluster formation by various eukaryotic SMC complexes. (**A**–**D**) Snapshots before and after DNA compaction by various SMC complexes of a doubly tethered DNA molecule. In schematics on the right, blue, yellow, and red represent DNA, biotin–streptavidin, and the SMC complex, respectively. (**E**) DNA cluster size over time (right) calculated based on the integrated fluorescence intensities of both the cluster and the full 48.5-kb λ-DNA in the left image after *Hs*–cohesin complex injection. Yellow circle indicates the compaction location. Exponential fit was used to measure DNA-compaction time (mean ± SD = 22.4 ± 2.0). (**F**) DNA-compaction time with and without ATP (mean ± SD; from left to right, *N* = 30, 10, 20, 10, 8, 10, 8, 20, 12, 20, 10, 12, and 20.). Deletion of Ycg1 from *Sc* condensin, or removal of the Mis4–Ssl3 loader from *Sp* cohesin and the NIPBL–MAU2 loader from *Hs* cohesin, abolished DNA clustering. Statistical significance was assessed using an unpaired *t*-test. (**G**–**J**) Snapshots of side-flow experiments before and after SMC addition [*N* = 10 for panels (G)–(J)]. (**K**–**N**) SMC/DNA cluster formation with labeled SMC complexes showing the colocalization of compacted DNA (blue) and SMCs (magenta). Average traces of DNA and SMC clusters (bottom; mean ± SEM). High correlation between the DNA and protein cluster formation was observed (*R* = 0.95, 0.97, 0.96, and 0.91; *N *= 7, 11, 13, and 13 for *Sc* condensin, *Sp* condensin, *Sp* cohesin, and *Hs* cohesin complexes, respectively.) Solid lines are the smoothed time traces using the Savitzky–Golay method with a moving window of 100 points.

**Figure 2.**
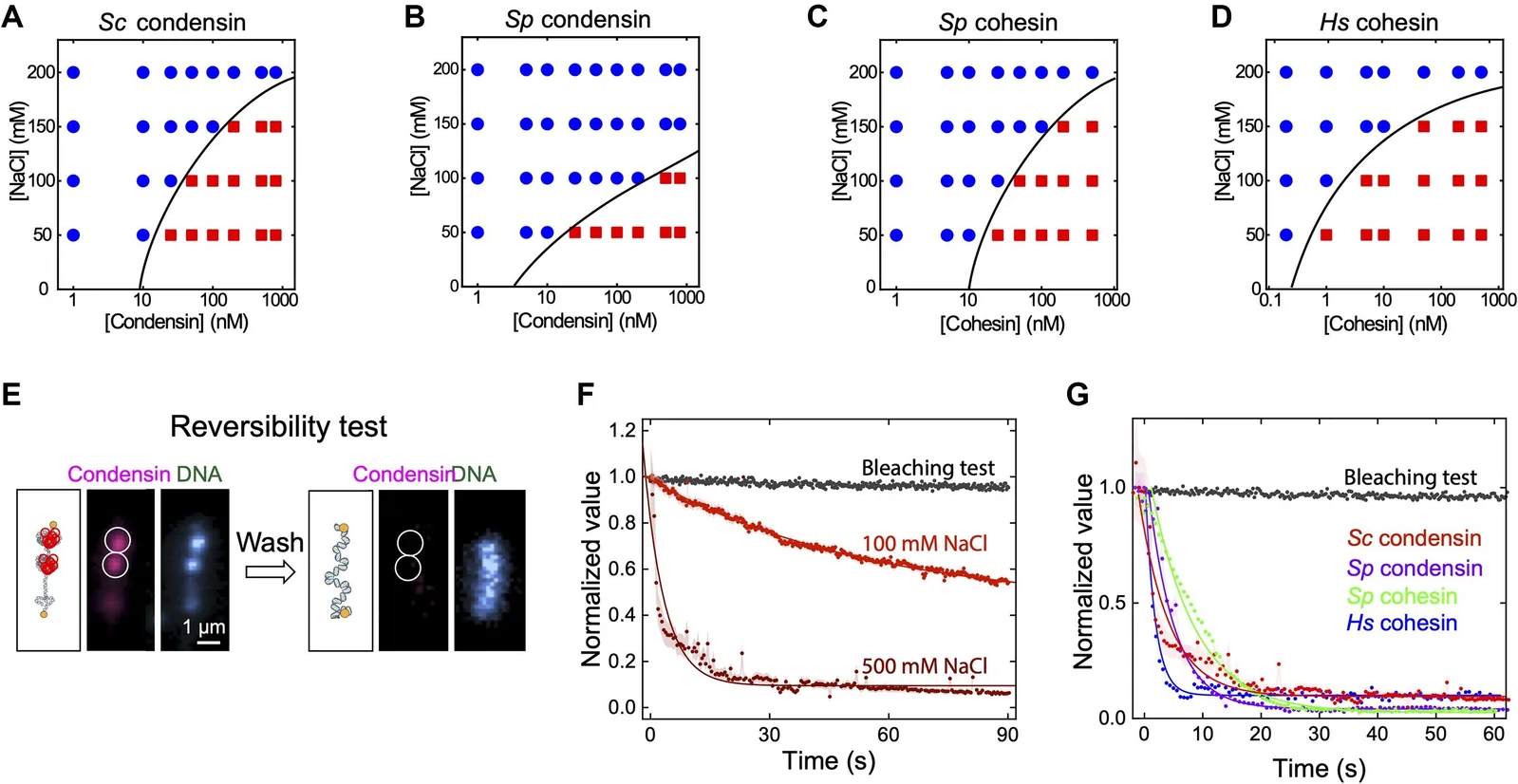
Electrostatic interaction-mediated SMC/DNA cluster formation. (**A**–**D**) Two-dimensional phase diagram of cluster formation induced by SMC complexes and λ-DNA at various salt (NaCl) and SMC complex concentrations. (**E**) Snapshots of the reversibility test of *Sc*-condensin complex/DNA clusters with washing in a high-salt buffer (before and after high-salt buffer wash). (**F**) Cluster intensities of *Sc* condensin complexes over time following washing (mean ± SEM; *N* = 60, 41, and 31; dissociation time constants (*τ*) = 1201 ± 31, 55.8 ± 0.8, and 5.2 ± 0.3 s for bleaching, 100 mM, and 500 mM NaCl, respectively). Dissociation time constants were obtained through exponential fitting. (**G**) Cluster intensities of *Sc* condensin, *Sp* condensin, *Sp* cohesin, and *Hs* cohesin complexes over time after washing with 500 mM NaCl buffer (mean ± SEM; *N* = 60, 27, 35, 31, and 77). Dissociation time constants (*τ*) obtained from exponential fitting were mean ± SD = 1201 ± 31, 1.1 ± 0.1, 4.5 ± 0.2, 7.6 ± 0.2, and 4.6 ± 0.2 s for bleaching, *Sc* condensin, *Sp* condensin, *Sp* cohesin, and *Hs* cohesin clusters, respectively).

**Figure 3.**
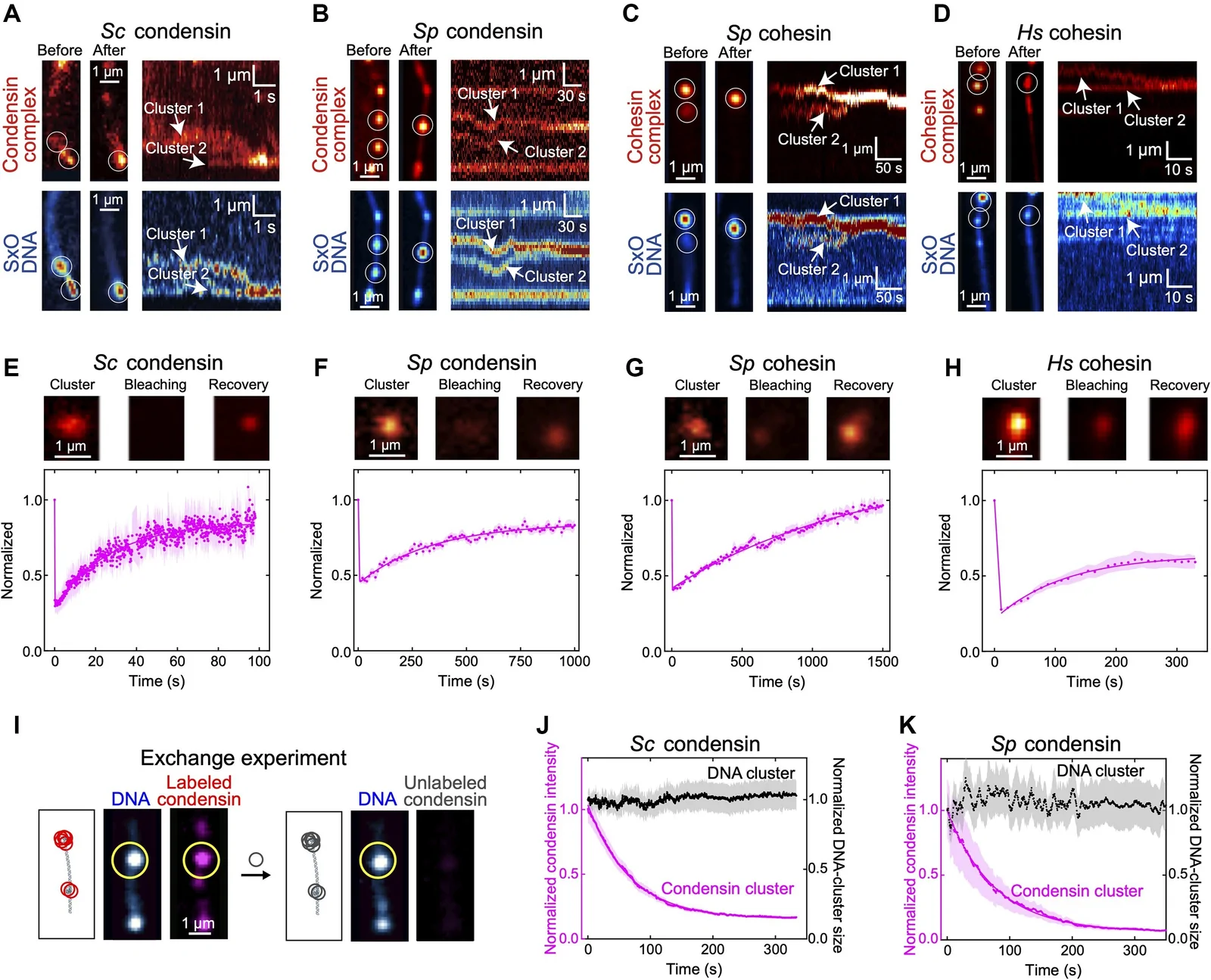
SMC complex forms liquid droplets along DNA. (A–D) Merging of two clusters, as monitored in the labeled SMC complexes (top) and DNA (bottom) channels. Left panels show two snapshots (before and after merging); right panels show fluorescence intensity kymographs [*N* = 158 (**A**), 22 (**B**), 65 (**C**), and 75 (**D**) merging events]. (E–H) FRAP experiments on SMC condensates. Snapshot images of a SMC condensate on DNA (top), before and after photobleaching, and after recovery. SMC cluster intensity versus time [bottom; mean ± SEM; the solid lines represent cumulative single-exponential fits; mean ± SD = 27.8 ± 0.9, 339 ± 20, 1,390 ± 109, and 115 ± 10 s for (**E**) *Sc* condensin (*N* = 13), (**F**) *Sp* condensin (*N* = 16), (**G**) *Sp* cohesin (*N* = 21), and (**H**) *Hs* cohesin (*N* = 7) complexes, respectively]. (I–K) Exchange of labeled condensin with unlabeled condensin complexes. Schematic and snapshots of labeled *Sc* condensin–DNA clusters before and after injection of unlabeled *Sc* condensin complexes (**I**). Condensin cluster intensities versus time upon injection of unlabeled condensin complex (magenta) (J, K). DNA-cluster sizes did not change [dissociation time: *τ* = mean ± SD = 63.5 ± 0.1 and 76.3 ± 0.4 s; for *Sc* condensin (*N* = 57) and *Sp* condensin (*N* = 10) clusters, respectively]. Magenta solid lines are exponential fits.

An electric syringe pump regulated buffer flow. Initially, T50 buffer (20 mM Tris, pH 7.5, and 50 mM NaCl) was introduced into the channels. To attach DNA to the PEG surface, a 100 μg/ml streptavidin solution was flushed through for 30 s, allowing it to bind to the biotin-PEG, followed by a T50 buffer rinse. A solution of double-biotinylated λ-DNA (100 pg/μl) in T50 buffer was then injected at 20 μl/min for 30 s, after which the flow rate was reduced to 3 μl/min for 20 min. SMF assays were performed in reaction buffer [50 mM Tris, pH 7.5; 50 mM NaCl; 2.5 mM MgCl₂; 0.5 mM TCEP; 0.5 mg/ml BSA; 10–50 nM SYTOX Orange (SxO)] ± 1 mM ATP, using >1 nM SMC complexes. Lower concentrations of SxO were used to reduce SxO-labeling artifacts. To observe the phase diagram in Fig. 2A–D, different NaCl and SMC-complex concentrations were applied. For labeled protein experiments (Figs 1K–N, 2E–G, and 3), an imaging buffer with 100 mM Tris (pH 7.5), 50 mM NaCl, 1 mM MgCl₂, 50 nM SxO, and an oxygen-scavenging system (2 mM trolox, 1% glucose, 300 μg/ml glucose oxidase, and 30 μg/ml catalase) was used.

For imaging SxO-stained DNA, 561-nm laser excitation was applied. Dual-color colocalization imaging of SxO-stained DNA and Atto648-labeled *Sc* condensin, Alexa647-labeled *Sp* cohesin/condensin, or JF646-labeled *Hs* cohesin complexes was achieved using alternating laser excitation at 561- and 642-nm wavelengths. A modified Nikon inverted epifluorescence microscope with a Nikon 100×/1.49 Apo TIRF oil immersion objective was employed for imaging. Image acquisition began right after introducing the reaction buffer, using highly inclined and laminated optical sheet (HILO) mode with temperature control (Okolab). An Andor iXon Ultra 897 CCD camera with a dual-emission image splitter (OptoSplit) captured images for dual-color experiments, and MetaMorph software recorded the single-molecule fluorescence images.

### FRAP experiments

After inducing the formation of SMC–DNA droplets in the microfluidic chamber using the protocol described above (excluding the oxygen-scavenging system), we photobleached the droplets by continuously exposing them to 642-nm laser excitation with consecutive 20-ms exposures, without any interval between exposures (Fig. 3E–H). To observe fluorescence recovery, we imaged the droplets at 10-s intervals between frames, using 20-ms laser exposures to minimize photobleaching of the labeled cohesin complexes (including the oxygen-scavenging system). The intensities were normalized against unbleached droplet intensities. The recovery time constant was determined through a single-exponential fit.

### Unlabeled/labeled-protein exchange experiment

To test whether labeled SMCs in a cluster can be replaced by unlabeled SMCs, we injected unlabeled SMC proteins onto the labeled SMC/DNA droplets in the microfluidic chamber (Fig. 3I–K). We imaged the droplets at 100 ms exposures using alternating laser excitation at 561- and 642-nm wavelengths. The normalized SMC cluster intensities were normalized by the initial cluster intensities. An exchange time constant was extracted by fitting the data to a single-exponential decay model. SxO-labeled DNA clusters were simultaneously analyzed to confirm that cluster size remained constant during the assay.

### Constructing a kymograph of DNA/protein images

To obtain a kymograph of a DNA image, fluorescence intensity profiles of DNA clusters on an immobilized λ-DNA were generated by summing the intensity values from 11 pixels along a line perpendicular to the extended DNA in each frame (Supplementary Figs S1, S3C–F, and S5G and H, and Fig. 3A–D). Background intensity was removed using two-dimensional median smoothing. To correct for intensity fluctuations such as bleaching, the profiles were normalized to the maximum value recorded during the measurements. A kymograph was then constructed from the normalized intensity profiles across all frames. For obtaining a kymograph of protein dynamics along an immobilized DNA, we constructed a kymograph without normalization to show protein intensities along a DNA (Supplementary Figs S1B, D, F, and H, S3C–F, H, S4B–E, and S5G and H, and Fig. 3A–D).

### Single-molecule imaging analysis

For kinetic analysis of DNA cluster formation, regions of interest (ROIs) for both the clustered area and the entire DNA area were defined using ImageJ (Fig. 1E, left). The total pixel intensities within these ROIs were measured for each frame, with background intensities subtracted by using low order 2D polynomial fitting and subtracted from the raw image. The size of the DNA in the clustered area (in kilobase pairs) was calculated by normalizing to the total DNA intensity and multiplying by 48.5 kb. The compaction time was obtained using an exponential fit. The kinetics of SMC cluster formation and release were measured by selecting the ROI where the labeled SMC-complex cluster colocalized with the DNA cluster formation, yielding intensity-time traces.

To analyze the bleaching steps of a single labeled SMC complex (Supplementary Fig. S2), the oxygen scavenging system (gloxy) was omitted to enhance bleaching of the fluorophores. To clearly measure a single-bleaching step, we bleached fluorophores labeled on the proteins that were non-specifically adsorbed onto the surface. Step sizes were determined by analyzing clusters using a step-finding algorithm based on a previously described method [31]. The labeling efficiencies for the SMC complexes used in the single-molecule assays were 82% (*Sc* condensin), 98% (*Sp* condensin), 90% (*Sp* cohesin), and 84% (*Hs* cohesin), all sufficiently high to avoid a large effect on the estimated stoichiometry.

To determine the compaction time, the time trace was smoothed using the Savitzky–Golay method with a 100-point moving window. To determine the time constant, a cumulative exponential function was fitted to the smoothed trajectory.

### AFM imaging and analysis

**Figure 4.**
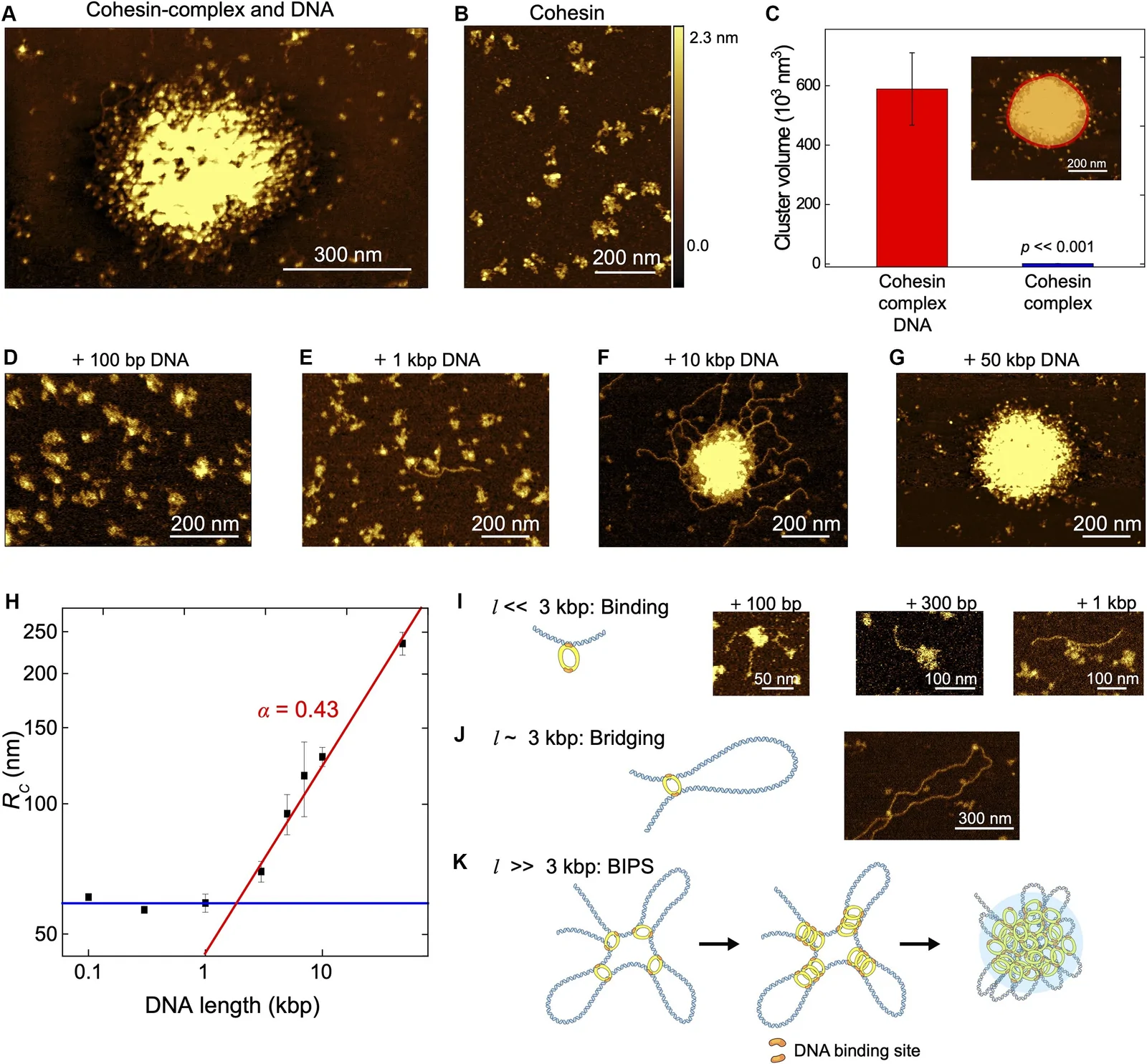
BIPS by *Hs* cohesin SMC complex. Representative AFM images of human cohesin–DNA clusters (**A**) and human cohesin complexes (**B**), and cluster volumes (**C**) (mean ± SEM, *N* = 14 and 73 clusters). (**D**–**G**) Representative AFM images with different DNA lengths ranging from 100 bp to 50 kb DNA. (**H**) Cluster size versus DNA length (mean ± SEM; *N* = 3040, 348, 44, 64, 19, 7, 56, and 14 for 0.1, 0.3, 1, 3, 5, 7, 10, and 48.5 kb DNA length, respectively). Log-log scale is shown. At short DNA length (*l*), the cluster size is constant (green line), while beyond a critical length ${{l}_C}{\mathrm{\ \gg \ }}3$ kb, the data exhibit a power law ${{R}_C}\sim{\mathrm{\ }}{{l}^\alpha }$ (blue line) with $\alpha = {\mathrm{\ }}0.43$ from a fit. (I–K) Schematic and representative images of clusters formed with short DNA (**I**) 100 bp, 300 bp, and 1 kb, (**J**) intermediate DNA (∼3 kb), and (**K**) long DNA (>3 kb). Examples include single cohesin complexes bound to short DNA fragments [panel (I)], DNA bridging by a single cohesin complex [panel (J)], and higher-order assemblies [panel (K)].

To visualize cohesin/DNA clusters, we combined 10 nM cohesin holocomplex and DNA (4 ng/μl) of various lengths [0.1, 0.3, 0.5, 1, 3, 5, and 10 kb (Thermo Fisher Scientific) and 48.5 kb (Promega)] in a reaction buffer [50 mM Tris (pH 7.5), 50 mM NaCl, 2.5 mM MgCl_2_, and 2 mM TCEP] in an E-tube, and then incubated the mixture for 5 min (see Figs 1J–M and 4). For Fig. 4H, to illustrate single cohesin-holocomplex-mediated DNA bridging, we mixed 1 nM cohesin holocomplex with 3 kb DNA (4 ng/μl) and incubated it briefly (10 s), using shorter DNA to limit large cluster formation. To test whether inter-DNA bridging by cohesin holocomplexes drives cluster formation, we mixed cohesin (1 or 10 nM) with 1 kb DNA molecules (3 or 30 ng/μl) in a buffer [50 mM Tris (pH 7.5), 50 mM NaCl, 1 mM MgCl_2_, and 1 mM TCEP] and incubated the samples for 10 min (Supplementary Fig. S8A–D). After incubation, the samples were deposited onto poly-l-ornithine (0.00001%, Millipore)-treated mica, rinsed with 3 ml of Milli-Q water, and dried using a nitrogen gun.

Atomic force microscopy (AFM) imaging of the dried samples was performed on a MultiMode 8 (Bruker) with a NanoScope V controller and NanoScope version 9.2 software [11, 16, 32]. Images of proteins and protein/DNA mixtures were recorded, and all measurements took place at room temperature. For processing dry AFM images, Gwyddion version 2.53 was used to remove background noise and to subtract plane background. Followed by blind tip estimation to assess tip shape and reconstruct the surface, we removed the broadening effects caused by tip convolution (widened images due to the nonzero AFM tip size).

To measure single protein volumes (Supplementary Fig. S6), we masked each protein in the images using Gwyddion, and then performed grain volume measurements for each masked protein. For cluster volume measurements, protein-bound DNA regions were manually delineated in Gwyddion, and their volumes (*V*) and areas (*A*) were obtained. For cluster-volume analysis of DNA/*Hs* cohesin complexes, a threshold was set at the mean +9σ of the *Hs* cohesin-only cluster-size distribution, corresponding to a one-sided probability of ∼1.1 × 10^−^¹⁹ under the assumption that cohesin-only cluster sizes are approximately normally distributed. The cluster size ${{R}_C}$ was measured by $\sqrt {A/\pi } $, where *A* is the cluster area (Fig. 4H and Supplementary Fig. S8A–D).

### Fluorescent tag ligands incubation and cell preparation for super-resolution imaging

The HCT116 CMV-OsTIR1 cell line used in previous studies [33, 34] and CRISPR/Cas9-derived knock-in cell lines were cultured at 37°C and 5% CO_2_ in DMEM (Gibco, 11995065) supplemented with 10% FBS (Gibco, 12483-020, Canada origin, Qualified) and antibiotics [100 U/ml penicillin and 100 μg/ml streptomycin (Gibco, 15140-122)]. All cell lines were tested for Mycoplasma contamination using the MycoStrip™—Mycoplasma Detection Kit (InvivoGen).

To generate the RAD21 C-term maid-halo knock-in repair template, the mClover3 sequence from pMK262 (RAD21-mAC Neo) (Addgene #140538) was replaced with the HaloTag sequence, amplified by PCR using the pENTR4-HaloTag plasmid (Addgene #29644) as a template.

To induce a double-strand break at the RAD21 locus, the RAD21 C-tag CRISPR plasmid (Addgene #140540) was used. HCT116 CMV-OsTIR1 cells cultured in 12-well plates were co-transfected with 1 μg of repair template and sgRNA-Cas9 plasmids using FuGene HD reagent (Promega, Cat#2311). At 48 h post-transfection, RAD21-mAID-Halo expressing cells were labeled with JF646-HaloTag ligand, sorted by fluorescence-activated cell sorting (FACSAria II), and seeded into 96-well plates for clonal selection. Genotypes of each monoclonal cell line were confirmed by western blotting.

### Super-resolution imaging

Cells were maintained at 37°C and 5% CO_2_ on glass-bottom dishes (Cellvis, D35-25-1.5-N) containing phenol red-free DMEM (Gibco, 21063-029) supplemented with 10% FBS (Gibco, 12483-020, Canada origin, Qualified) and antibiotics [100 U/ml penicillin and 100 μg/ml streptomycin (Gibco, 15140-122)]. For imaging of HaloTag-labeled RAD21, JF549-HaloTag ligand (Janelia Fluor® HaloTag Ligands, Promega, GA111) dissolved in dimethyl sulfoxide was added to culture medium at a final concentration of 100 nM. Cells were incubated for 10 min at 37°C and 5% CO_2_ to facilitate ligand binding to the HaloTag. Excess unbound ligand was removed by replacing the medium with fresh culture medium and incubating for an additional 10 min; this washing step was repeated twice. For fixation, cells were washed with phosphate buffered saline (PBS) and then fixed with 4% paraformaldehyde for 10 min at room temperature. After fixation, paraformaldehyde was removed, and cells were washed three times with PBS (5 min each wash) to ensure complete removal.

All super-resolution imaging data in this study were acquired using a custom-built microscope based on the Nikon Ti2e microscope body equipped with HILO illumination. In HILO illumination, the incident angle is set slightly below the critical angle that induces total internal reflection (TIR), resulting in a thin excitation light sheet capable of illuminating deeper regions within the sample. Cells were illuminated sequentially with 405 and 561 nm lasers (CUBE; Coherent) for excitation. Images were acquired with an EMCCD camera (Andor, iXon Ultra 888) and processed using NIS-Elements software. Subsequent image analysis was performed using ImageJ scripts.

**Figure 5.**
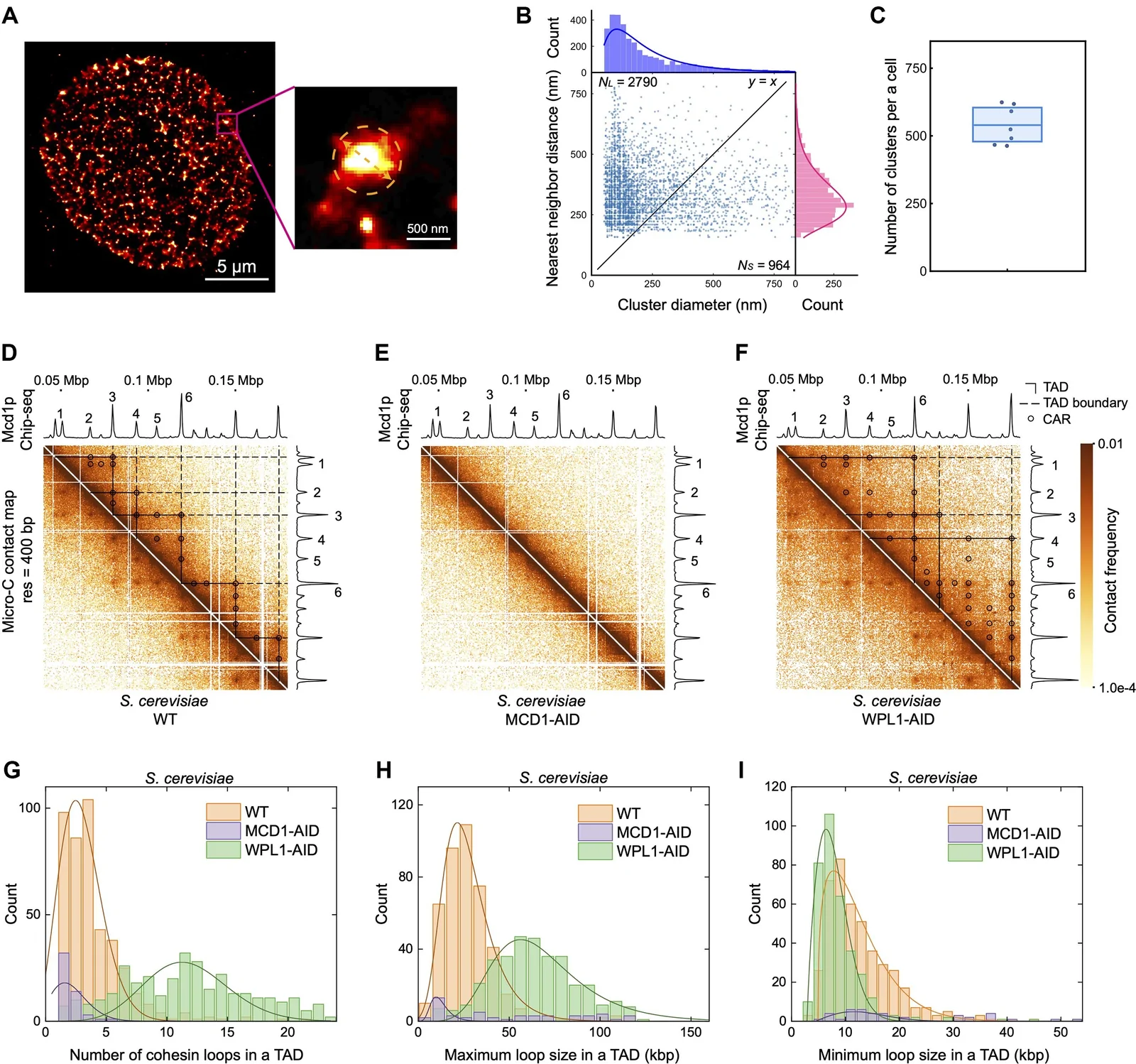
Super-resolution images and Micro-C data show cohesin condensates. (**A**) Representative super-resolution image of endogenous cohesin labeling in HCT116 cells (number of cells = 8). Magnified inset highlights an individual cohesin condensate. (**B**) Scatter plot of cohesin condensate diameters versus nearest-neighbor distances between condensates. The top and right panels show the distributions of condensate diameters [median (mode) ± SD = 164 (86) ± 231 nm] and nearest-neighbor distances [median (mode) ± SD = 321 (282) ± 110 nm; number of condensates = 3754], respectively. Solid lines represent log-normal fits. The *y* = *x* line indicates that most condensates (*N* = 2790) had nearest-neighbor distances greater than their diameters, whereas a minority (*N* = 964) exhibited shorter nearest-neighbor distances. (**C**) Quantification of the number of cohesin condensates per cell (mean ± SD = 539 ± 65; number of cells *n* = 7 cells). The range of the box plots corresponds to mean ± SD, with the central line representing the mean. Micro-C contact maps (400 bp resolution) and mcd1p ChIP–seq for *S. cerevisiae* WT (**D**) mcd1p-depleted (**E**), and wpl1p-depleted (**F**) cells (40–180 kb region, chromosome II) [37]. (**G**) Distributions of the number of cohesin-organized loops per topologically associating domain (TAD) in WT, mcd1Δ, and wpl1Δ cells (mean ± SD = 3.4 ± 2.6, 1.5 ± 0.9, and 12.3 ± 6.8 loops; *N* = 1331 loops from 432 TADs, *N* = 78 loops from 51 TADs, and *n* = 2721 loops from 348 TADs for WT, mcd1Δ, and wpl1Δ cells, respectively). Poisson fits shown (solid lines). (**H**) Distribution of maximum loop sizes per a TAD (mean ± SD = 28 ± 17, 50 ± 37, and 63 ± 24 kb; *N* = 432, 51, and 348 TADs for WT, mcd1Δ, and wpl1Δ cells, respectively). Gumbel fits shown (solid lines). (**I**) Distribution of minimum loop sizes per a TAD (mean ± SD = 12.4 ± 6.13, 33 ± 29, and 53 ± 5.1 kb; *n* = 432, 51, and 348 parent TADs for WT, mcd1Δ, and wpl1Δ cells, respectively). Weibull fits shown (solid lines).

For dSTORM imaging of JF549-HaloTag ligand, cells were illuminated using both 405- and 561-nm laser. For each dataset, 12 000–15 000 frames are acquired at a temporal resolution of 30 ms per frame. Imaging was performed in an imaging buffer containing 10% glucose, 100 mM MEA (Sigma, 30070), and 1% gloxy solution [56 mg/ml glucose oxidase (Sigma, G2133-250KU), 500 U/ml catalase (MP Biomedicals, 02100402-CF) in PBS] in PBS. Acquired images were then analyzed and reconstructed into super-resolved images using the image J plugin ThunderSTORM [35] (Fig. 5A–C and Supplementary Fig. S9).

### Analysis of cohesin cluster size in super-resolution images

To quantify the size of cohesin clusters, contours were extracted using the findContours function in OpenCV (Fig. 5 and Supplementary Fig. S9). An optimal threshold was determined using Otsu’s method, after which cluster boundaries were identified. For each detected contour, the minimum enclosing circle was computed to estimate the radius. Clusters with an area smaller than 2 pixels were considered noise and excluded from further analysis.

To assess the spatial proximity of cohesin clusters, super-resolution images were acquired from seven STORM-imaged cells. Super-resolution reconstruction was performed using ThunderSTORM. The resulting images were denoised by applying a Gaussian filter with a sigma of 1 pixel. Local intensity peaks were subsequently identified to extract the spatial coordinates of individual cohesin clusters. These coordinates were then structured into a KDTree for efficient spatial querying. Using this KDTree, the distance to the nearest neighbor and the mean distance to the five nearest neighbors were calculated for each cluster (Fig. 5B).

### Multi-TADs analysis on Hi-C/Mi-C data

To confirm cohesin-mediated loop formation, ChIP-seq and Micro-C data were analyzed. Initially, peaks from RAD21 or mcd1p (the yeast homolog of RAD21) ChIP-seq datasets were identified (Fig. 5D–F and Supplementary Fig. S9). The association between two ChIP-seq peaks was then evaluated by determining the proximity of Hi-C dots connecting two loci. Alternative approaches such as ChIA-PET or Hi-C exist; however, currently available datasets do not surpass the resolution and loop detectability provided by Hi-C data used in this study.

RAD21/mcd1p ChIP-seq data were analyzed using custom Python scripts. For *H. sapiens* analyses, RAD21 ChIP-seq data from GM12878 WT cells were employed (Supplementary Fig. S9). Cohesin-bound peaks were designated as cohesin-associated regions (CARs) following the nomenclature introduced by the previous study [36]. Prominence threshold of 1.66 and distance 30 kb from the previous peak, are used as filtering condition. For *S. cerevisiae*, publicly available datasets from GEO were utilized (accession code GSM4577764) [37] (Fig. 5D–F). These datasets include ChIP-seq signals from WT cells and cells where mcd1p or wpl1p (the yeast homolog of WAPL) were degraded via the auxin-inducible degron (AID) system. Detected peaks were filtered by prominence threshold of 5, and minimum distance 3,200 bp from the previous peak.

Micro-C datasets from the Hffc6 *H. sapiens* cell line (accession code 4DNFI9FVHJZQ) [38] and *S. cerevisiae* (accession code GSE151553) [37] were processed and analyzed using Cooltools [39]. Due to the high-resolution requirement for distinguishing ChIP-seq peaks from adjacent signals, Micro-C dots for *H. sapiens* Hffc6 cells were detected at resolutions of 5, 10, and 25 kb, using window sizes of 15, 9, and 5, respectively. For yeast, dots were identified at resolutions of 400, 800, and 1600 bp, with kernel size of 13, 9, 5. 800, 1600 bp resolution has gone through additional detection with kernel size 7.

Contacts between CARs were evaluated based on proximity to detected Micro-C dots, applying the range tolerance of and 30 kb and 3200 bp to *H. sapiens* and yeast, respectively. Cohesin loops (CAR-CAR interactions) were grouped into single TADs if they met either of two criteria: [1] loops shared a common anchor region, or [2] a larger loop encompassed both CARs. Visual inspection confirmed this method accurately identified looped domains (TADs) and provided superior boundary detection compared to conventional insulation-based methods (e.g. as implemented in Cooltools [39]).

## Results

### ATP-independent DNA cluster formation

We visualized DNA-bound clusters formed by a variety of eukaryotic SMC protein complexes—including *Sc* condensin (Smc2–Smc4–Brn1–Ycg1–Ycs4), *Sp* condensin (Cut14–Cut3–Cnd2–Cnd1–Cnd3), *Sp* cohesin (Psm1–Psm3–Rad21–Psc3 with loader Mis4–Ssl3), and *Hs* cohesin (SMC1–SMC3–RAD21–STAG1 with loader NIPBL–MAU2)—under *in vitro* conditions (Fig. 1A–D and Supplementary Movie S1 for *Sp* condensin). λ-DNA (48.5 kb) was immobilized on a PEG-coated surface using a biotin–streptavidin linkage, whereupon it was stained with SxO, and SMC complexes were introduced at concentrations exceeding 1 nM [16, 40]. Remarkably, all tested SMC complexes formed DNA-associated clusters both in the presence and absence of ATP, indicating that this condensation is ATP independent. Previous single-molecule studies of ATP-dependent loop extrusion were performed at low protein concentrations (<1 nM) [5–8], a regime chosen to resolve individual extruding complexes; here, we examined the higher concentration range, in which SMC complexes robustly cluster DNA independently of ATP.

To quantify the kinetics of cluster formation, we measured the fluorescence intensity over time at defined cluster regions upon following addition of the *Hs* cohesin to the DNA on the glass slides (Fig. 1E; see the “Materials and methods” section). The fluorescence signal increased exponentially and reached a plateau within ∼40 s. The compaction time constant (*τ*) obtained from the exponential fit was 22.4 ± 2.0 s. Control reactions without SMC complexes showed no detectable clustering. By contrast, both condensin (*Sc* and *Sp*) and cohesin (*Sp* and *Hs*) induced DNA compaction under both ATP-present and ATP-free conditions (Fig. 1F). The similarity in compaction kinetics under both conditions suggests that this behavior is independent of ATP hydrolysis and mechanistically distinct from canonical loop extrusion. Moreover, under side-flow conditions, the clusters remained stable and did not show any visible DNA loop extrusion (Fig. 1G–J and Supplementary Movie S2). However, we found that clustering depends on these DNA-binding components—the Ycg1 subunit of *Sc* condensin and the cohesin loaders Mis4–Ssl3 (*Sp*) and NIPBL–MAU2 (*Hs*): in their absence, no DNA clustering was observed (Fig. 1F and Supplementary Fig. S1A–C).

To investigate how many SMC complexes assemble into DNA-bound clusters, we applied fluorescently labeled SMC complexes to doubly tethered λ-DNA. SMC complexes were observed to colocalize with the DNA clusters (Fig. 1K–N and Supplementary Movie S3 for *Hs* cohesin), with minimal signal detected outside of the cluster regions. A concurrent increase in fluorescence intensities of both DNA and SMC components was consistently observed during cluster formation (Fig. 1K–N and Supplementary Fig. S1), and the intensity changes were highly correlated (Pearson’s correlation *R* > 0.9) across all four SMC complex types tested. To estimate the number of SMC complexes per cluster, we compared the total fluorescence intensity of individual clusters with the average intensity of single SMC complexes, as determined from bleaching steps of non-specifically absorbed individual molecules (Supplementary Fig. S2). Based on this approach, we estimated the number of SMC complexes per cluster as 1080 ± 860 for *Sc* condensin, 254 ± 106 for *Sp* condensin, 405 ± 316 for *Sp* cohesin, and 777 ± 941 for *Hs* cohesin (mean ± SD unless otherwise noted; Supplementary Fig. S2G–J). The labeling efficiency for each SMC complex used in the single-molecule assays ranged from 82% to 98%, sufficiently high to minimize its effect on the estimated stoichiometry. These data indicate that SMC/DNA co-condensates are composed of many hundreds of protein complexes and suggest a cooperative assembly mechanism consistent with collective multivalent interactions.

### Phase diagram of SMC/DNA clustering

The SMC–DNA clusters exhibited hallmark features of biomolecular condensation, as evidenced by their dependence on both SMC protein concentration and ionic strength [41]. A two-dimensional phase diagram revealed that cluster formation was promoted at higher SMC concentrations (>100 nM) and lower NaCl concentrations (<150 mM) (Fig. 2A–D), implicating electrostatic interactions between SMC complexes and DNA as key drivers of condensation. The precise transition thresholds for clustering varied between SMC complex types, indicating varying DNA-binding affinities across complexes. Inclusion of a molecular crowding agent to mimic physiologically relevant conditions further lowered the critical concentration thresholds for both protein and salt, enhancing SMC–DNA interactions (Supplementary Fig. S3A and B).

Notably, robust condensate formation was observed for all four SMC complexes at physiological salt conditions (∼150 mM NaCl) and protein concentrations comparable to estimated intracellular levels (e.g. >1 μM for yeast condensin and cohesin; ∼330 nM for *H. sapiens* cohesin) [42–46]. Although condensation was robust in every case, the saturation boundaries differed reproducibly among species and complexes (Fig. 2A–C). These shifts are consistent with known differences in the complexes’ DNA-binding modules: the human cohesin HAWK subunit STAG binds dsDNA with tens-of-nanomolar affinity [47], whereas the budding-yeast condensin Ycg1–Brn1 anchor binds more weakly (micromolar range [48]), and the fission-yeast complexes show ATP-independent but strongly salt-sensitive DNA binding [28]. The protein concentrations at which each complex condensed broadly track this order, with the more avid DNA binder (*Hs* cohesin) condensing at the lowest concentration. We regard these as qualitative, module-level differences rather than directly comparable constants, as BIPS depends on multivalent inter-DNA bridging rather than on monovalent DNA affinity alone; differences in DNA-contact valency, HEAT-repeat flexibility, and salt sensitivity are all likely to contribute. Because these concentrations overlap those at which loop extrusion operates in cells, BIPS and loop extrusion likely act together rather than as mutually exclusive modes of SMC function. To assess reversibility—a hallmark of equilibrium biomolecular condensation—we increased the salt concentration or removed SMC proteins from the buffer. Both treatments led to rapid dissolution of DNA–SMC clusters (Fig. 2E–G and Supplementary Fig. S3C–F). Exposure to 500 mM NaCl dissociated all complexes within 10 s (Fig. 2F and G and Supplementary Movie S4 for *Sp* cohesin). Reintroduction of SMC proteins restored clustering, further confirming the dynamic, reversible nature of SMC–DNA condensation (Supplementary Fig. S3G–I and Supplementary Movie S5 for *Hs* cohesin).

To assess whether weak hydrophobic interactions contribute to the condensate formation, we treated *in vitro* samples with 1,6-hexanediol—an aliphatic alcohol known to disrupt weak protein–protein and protein–nucleic acid interactions (Supplementary Fig. S4A) [49]. This reagent is commonly used to distinguish liquid-like biomolecular condensates from more solid-like or gel-phase assemblies, as it dissolves dynamic droplets while sparing rigid structures [49, 50]. Upon 1,6-hexanediol treatment, we observed rapid disruption of SMC–DNA condensates (*τ* = mean ± SD = 3.0 ± 0.1, 8.3 ± 0.5, 3.3 ± 0.3, and 6.7 ± 0.3 s for *Sc* condensin, *Sp* condensin *Sp* cohesin and *Hs* cohesin, respectively), consistent with previous studies on condensation systems (Supplementary Fig. S4B–F and Supplementary Movie S6 for *Sp* cohesin) [16]. Interestingly, a subset of SMC complexes remained bound to DNA even in the presence of 1,6-hexanediol, suggesting that not all interactions were disrupted. This residual binding indicates that non-hydrophobic forces—such as electrostatic interactions—also contribute to SMC–DNA association, in agreement with salt-dependent reversibility observed in earlier experiments (Fig. 2). Some SMC complexes will have topologically entrapped DNA under our experimental conditions, which would render them resistant to removal by 1,6-hexanediol [16, 51, 52].

### Dynamic properties of SMC/DNA condensates

The SMC/DNA condensates exhibited dynamic behaviors consistent with liquid-like properties. When multiple clusters formed along a single DNA molecule, neighboring condensates on the same DNA frequently coalesced into larger clusters [Fig. 3A–D, Supplementary Fig. S5A–F, and Supplementary Movie S7 for *Sp* cohesin (A) and *Hs* cohesin (B)]. This merging occurred simultaneously for both the DNA and SMC signals, suggesting that the condensates behave as cohesive, surface-tension-driven co-assemblies. All four tested SMC complexes exhibited this coalescence behavior, supporting a general property of SMC-mediated condensation.

To assess molecular turnover within these condensates, we performed fluorescence recovery after photobleaching (FRAP) experiments. Fluorescently labeled SMC complexes within DNA-bound droplets were photobleached, and recovery of fluorescence was monitored over time (Fig. 3E–H). We observed rapid fluorescence recovery, indicating continuous exchange of SMC complexes between the condensed phase and the surrounding solution. This dynamic turnover is a hallmark feature of liquid biomolecular condensates.

We further validated this exchange behavior by replacing labeled SMC complexes with unlabeled counterparts. Upon injection of unlabeled SMC complexes into pre-formed labeled clusters, the fluorescence intensity of the labeled SMC clusters (Fig. 3I) decreased, while the DNA cluster size (marked by SxO staining) remained unchanged. Since we used the same laser excitation as in the photo-bleaching control (Fig. 2F and G), the rapid decrease cannot be attributed to photo-bleaching. Both *Sc* and *Sp* condensin clusters showed efficient replacement with their unlabeled forms [Fig. 3 J and K, Supplementary Fig. S5G and H, and Supplementary Movie S8 for *Sc* condensin (A) and *Sp* condensin (B)], confirming that the SMC proteins within the condensates are dynamically exchanged with those in the bulk. Collectively, these results demonstrate that SMC/DNA condensates behaves as liquid-like droplets—highly dynamic, reversible assemblies with rapid internal molecular turnover.

### DNA bridging triggers SMC/DNA clustering

To investigate the mechanism of cluster formation, we performed AFM on mixtures of λ-DNA and *Hs* cohesin [53]. Robust cluster formation was observed only when both DNA and *Hs* cohesin were present (Fig. 4A). *Hs* cohesin alone did not form large or organized clusters (Fig. 4B and C). AFM imaging showed *Hs* cohesin bound extensively along the DNA, resolving into discrete, well-separated clusters distributed across most DNA regions (Fig. 4A), consistent with localized cohesin–DNA interactions at this concentration. The volume of DNA-bound clusters was substantially greater than that of background particles in cohesin-only samples, with the two volume distributions largely non-overlapping (Fig. 4C and Supplementary Fig. S6). This indicates that DNA–cohesin interactions—not cohesin–cohesin self-oligomerization—drive cluster formation. Consistent with an electrostatic basis for these interactions, the salt-dependent phase behavior (Fig. 2A–D) and salt-mediated dissociation (Fig. 2G) suggest that contacts between the negatively charged DNA backbone and cohesin contribute substantially to condensation.

We next examined whether DNA bridging underlies cohesin-mediated clustering. Cluster formation by *Hs* cohesin was strongly dependent on DNA length, with a threshold within ~3 kb required to initiate condensation (Fig. 4D–H and Supplementary Fig. S7). DNA fragments shorter than this failed to support clustering, consistent with the inability of stiff short DNA to spontaneously form entropically stable loops through thermal fluctuations. Above this threshold, cluster size scaled with DNA length according to a power law ${{R}_C} = {{l}^\alpha }$, where $\alpha = 0.43 \pm 0.02$, consistent with a bridging-induced condensation mechanism (Fig. 4F–H) [16, 22]. Furthermore, extended DNA substrates gave rise to rosette-like multi-loop architectures (Fig. 4F and Supplementary Fig. S7D and E). This scaling deviates from that expected for simple DNA–protein binding and instead suggests a cooperative growth process initiated by DNA bridging. Indeed, AFM directly visualized individual *Hs* cohesin, *Sc* condensin, and *Sp* cohesin complexes each contacting two DNA segments simultaneously (Fig. 4J and Supplementary Fig. S7F and G), consistent with their multiple DNA-binding domains and with a bridging-competent DNA length near 3 kb. Owing to the weaker DNA-binding affinities of the other SMC complexes (Fig. 2A–C), visualizing their SMC–DNA clusters was difficult: the high protein concentrations required to form clusters produced a high protein/DNA background that obscured individual features. As a complementary approach, we therefore removed the DNA-binding components required for chromosomal engagement—the Ycg1 subunit of *Sc* condensin and the cohesin loaders Mis4–Ssl3 (*Sp*) and NIPBL–MAU2 (*Hs*)—and assessed the resulting loss of multivalent bridging in BIPS (Fig. 1F).

This remarkable length dependence and cooperative scaling behavior are well described by the BIPS model, in which initial DNA bridging events nucleate condensate formation, followed by recruitment of additional SMC complexes that stabilize and expand the cluster (Fig. 4K). The power-law scaling of cluster size with DNA length reveals underlying properties of the condensation. A power-law scaling with alpha = 1/3 is generally associated with fully collapsed globular polymer conformations, while alpha = 3/5 is associated with Flory ideal polymer [54, 55]. We found that the experimentally deduced exponent 0.43 falls between 1/3 (globular collapse) and 3/5 (flory ideal polymer). Notably, this value is very close to a previous result of 0.45 obtained for *Sc* cohesin, which forms clusters by bridging DNA via two DNA binding interfaces [16].

This intermediate exponent is consistent with the positive feedback central to the BIPS model. An initial bridging event raises the local DNA density, which in turn elevates the probability of further bridging—both by increasing the collision frequency between DNA segments and by lowering the entropic cost of forming additional crosslinks. This framework treats SMC complexes as multivalent DNA binders and DNA as a flexible polymer whose conformation is progressively compacted as bridges accumulate. Such density-dependent reinforcement can drive robust condensate growth once nucleation occurs, in line with our observations [22–25, 56]. Together, these findings highlight DNA bridging as a critical determinant of SMC-mediated cooperative condensation and support a mechanistic framework in which BIPS contributes to chromosomal organization.

For the DNA-length-dependence experiments, we used a low DNA concentration to isolate cluster formation within a single DNA molecule; however, at higher DNA concentrations, clusters induced by inter-DNA bridging became detectable even with 1 kb DNA, indicating inter-DNA condensation (Supplementary Fig. S8). To promote inter-DNA interactions, we varied the cohesin and DNA concentrations using 1 kb DNA (Supplementary Fig. S8A–D). At low concentrations (1 nM cohesin, 3 ng/μl DNA), larger clusters were rarely observed (Supplementary Fig. S8A), whereas at higher concentrations (10 nM cohesin, 30 ng/μl DNA) they became more frequent (Supplementary Fig. S8B). Because these clusters contain multiple DNA molecules, they are likely formed through multi-DNA bridging. They were nonetheless smaller than those formed with longer DNA (>250 nm for λ-DNA with cohesin; Fig. 4H), suggesting that the higher local DNA density along a longer molecule provides more opportunities for bridging. Using SMF microscopy, we further observed that two separate DNA molecules could undergo cohesin-mediated zippering (Supplementary Fig. S8E): once the two molecules were bridged at an initial contact point, zippering proceeded from that point toward the ends of the molecules, supporting BIPS through inter-DNA interactions (Supplementary Fig. S8F).

### Super-resolution images and Micro-C data show cohesin condensates

To assess whether the cohesin clusters observed *in vitro* by AFM are also present in living cells, we performed super-resolution imaging of endogenously tagged *H. sapiens* cohesin in HeLa cells (Fig. 5 and Supplementary Fig. S10) [33, 57, 58]. Cohesin formed discrete nuclear clusters with a mode (most probable value) diameter of 86 ± 231 nm (mode ± SD) (Fig. 5A and B), suggesting that cohesin is not diffusely distributed but instead locally concentrated in higher-order assemblies. Notably, 74.3% of condensates exhibited nearest-neighbor distances (mode ± SD = 282 ± 110 nm) that were significantly larger than their diameters (86 nm), indicating that cohesin condensates are dispersed and largely isolated (Fig. 5B). Quantitative analysis revealed 539 ± 65 clusters per nucleus under steady-state conditions, with a substantial (>27.7%) subset exceeding 300 nm in diameter—indicative of larger structures (Fig. 5C). Notably, cluster-like cohesin assemblies were reported in earlier studies [59]. These results support the existence of cohesin condensates *in vivo*, which we hypothesize are due to a condensation-based mechanism.

To investigate the contribution of cohesin clustering to 3D genome architecture, we re-analyzed previously published Micro-C contact maps from HFFc6 wild-type cells in combination with RAD21 ChIP-seq data from cells [36, 38] (Supplementary Fig. S10). The 11.5–13 Mb region on chromosome X exhibited prominent loop-rich domains. Many loop anchors colocalized with ChIP–seq peaks, defining candidate CARs that appear to interact in a lattice-like configuration. Given that a single cohesin complex typically forms one loop, we hypothesized that clusters of cohesin complexes could mediate the formation of multi-loop domain architectures. To test this, we identified the large TADs and quantified the number of internal loops within each. Loops were defined as Hi-C loop calls that colocalize with cohesin ChIP-seq peaks. For example, Supplementary Fig. S10A illustrates an ∼1 Mb TAD containing ∼17 internal loops nested within a larger loop (spanning loop anchor loci 2–8, coincident with the TAD’s boundaries). Across multiple loci, each TAD spanned 0.3–1.7 Mb—set by its largest (outermost) loop (Supplementary Fig. S10B)—and contained 15–25 nested loops (Supplementary Fig. S10C). Given the ∼2-kb resolution of Hi-C, this likely represents a lower bound, as additional smaller loops are likely to remain undetected. These results support a model in which cohesin clustering facilitates the formation of hierarchical nested loop domains in *H. sapiens* cells.

To further investigate chromatin architecture and the impact of cohesin accumulation on chromatin topology, we also analyzed previously published ultra-high-resolution Micro-C maps (∼100 bp resolution) from mitotic *S. cerevisiae* [37]. We compared wild-type, Mcd1p-depleted (a core cohesin subunit), and Wpl1p-depleted (a cohesin unloader) strains, where depletion was achieved with a auxin-inducible degron system. In wild-type cells, multiple loops were detected within each TAD (mean ± SD = 3.4 ± 2.6 loops; Fig. 5D and G), with a maximum loop size of 28 ± 17 kb (Fig. 5H) within each TAD. Loop anchors predominantly coincided with cohesin ChIP-seq peaks. Mcd1p depletion, however, significantly reduced the number of nested loops (1.5 ± 0.9 loops; Fig. 5E and G) within each TAD and disrupted the lattice-like interaction patterns, although the remaining loops exhibited an increased maximum size (50 ± 37 kb; Fig. 5H). In contrast, Wpl1p depletion resulted in a marked increase in both the number and complexity of internal loops (12.3 ± 6.8 loops; Fig. 5 F and G), with an enlarged maximum loop size (63 ± 24 kb; Fig. 5H). Moreover, in Wpl1p-depleted cells, smaller TADs appeared to fuse into larger domains, consistent with the formation of expanded cohesin clusters (Fig. 5F). Increased cohesin residence time, altered loop-extrusion dynamics, or secondary chromatin reorganization may also contribute to this domain enlargement.

We further analyzed the loop size distributions within TADs in these data. The maximum loop size per TAD was 28 * ± * 17 kb (mean ± SD), with the minimum loop size per TAD averaging 12 * ± * 6 kb in WT cell (Fig. 5H and I). Notably, the observed minimum loop size is consistent with a model in which thermally driven DNA fluctuations enable bridging and nucleate condensation. However, loops of this scale could, of course, also arise from loop extrusion. Theoretically, the small loop size scale (∼10 kb) can be generated by bridging induced by thermal fluctuation: adopting a persistence length of native yeast chromatin of ${{l}_p} \approx 30\ {\mathrm{nm}}$ or about 460 bp [60] given that each nucleosome core is wrapped by 147 bp of DNA, and the linker DNA between nucleosomes is ~15 bp, the stable minimal loop size can be analytically derived from free energy minimization: $\frac{F}{{{{k}_B}T}} = \frac{{2\epsilon {{l}_p}}}{l} + c\ \mathrm{ log}( {\frac{l}{{{{l}_p}}}} )$ (Ryu *et al*. and Park *et al*. [16, 22]), yielding a bridged loop size $l = \frac{{2\epsilon {{l}_p}}}{c}{\mathrm{\ }} = {\mathrm{\ }}9813{\mathrm{\ bp}}$, where *c* characterizes the contact probability of two segments in a polymer (*c* = 1.5 for an ideal random walk) [55] and the shape factor, *ϵ* = 16 (tear-drop shape) [56, 61]. The observed minimal loop sizes (∼10 kb) are consistent with this theoretical prediction, suggesting that bridged loops can in principle be formed by cohesin bridging next to loop extrusion. Multiple bridged loops organized by several cohesin complexes may not only promote domain compaction but also may facilitate the formation of multi-loop structures. The observed increases in both TAD size and the number of shorter loops upon Wpl1p depletion support the idea that BIPS contributes to cohesin-mediated multi-loop chromatin organization, as increased cohesin loading leads to the formation of larger condensates.

## Discussion

Our findings demonstrate that phase condensation via BIPS is a conserved feature of eukaryotic SMC complexes across species and SMC types, complementing their established role in DNA-loop extrusion. We demonstrate that BIPS occurs in *Sc* condensin, *Sp* condensin, *Sp* cohesin, and *Hs* cohesin—extending this mechanism across species and SMC subtypes. Previous reports had documented BIPS in *Sc* cohesin [16]; our results show it is a broadly conserved feature. Notably, we observed that *Hs* cohesin also exhibits DNA-length dependent cohesin/DNA condensate formation requiring DNA–cohesin–DNA bridging, similar to the yeast cohesin behavior [16]. These results highlight the dual functionality of SMC complexes, which integrate DNA loop extrusion and phase condensation to dynamically organize genome architecture.

The structural features of SMC complexes provide a mechanistic rationale for BIPS. Cryo-EM studies have revealed multiple DNA-binding interfaces across SMC subunits—including Ycg1 and Ycs4 in *Sc* condensin, and NIPBL and STAG1 in *Hs* cohesin—as well as DNA contacts at the hinge and ATPase head domains [4, 12, 59, 60, 61]. Consistent with this, complexes lacking a DNA-binding subunit failed to cluster DNA (Fig. 1F and Supplementary Fig. S1A–C), as predicted by the BIPS mechanism. These multivalent DNA-binding capabilities enable SMC complexes to bridge noncontiguous chromatin segments, driving local increases in protein concentration that nucleate condensate formation. The resulting structures display dynamic properties characteristic of biomolecular condensates, including FRAP and sensitivity to 1,6-hexanediol (Figs 2 and 3, and Supplementary Fig. S4).

The ability of SMC complexes to form DNA-associated condensates has broad implications for nuclear organization *in vivo*. Super-resolution imaging and high-resolution chromatin conformation assays, including Micro-C, reveal that cohesin and condensin form discrete nuclear clusters (condensates) during both interphase and mitosis (Fig. 5), consistent with biomolecular condensation observed *in vitro* [38, 58, 62].

These condensates are likely to scaffold higher-order chromatin structures. Micro-C data indicated hierarchical TAD organization, often featuring relatively short, nested loops anchored at cohesin-enriched sites (Fig. 5D and I). The accumulation of cohesin on chromatin supports the assembly of multi-loop domains through DNA bridging. Importantly, loop extrusion accounts for the formation of individual anchor-defined loops but not for their aggregation into multi-loop domains; the co-clustering of loops we observe is consistent with an additional bridging-driven step, in which cohesin condensates gather multiple loop anchors into a single higher-order structure. Such multi-loop domains emerge under different anchoring logic across species—positioned by CTCF in mammals but forming at CARs in budding yeast, which lacks CTCF—suggesting that BIPS-mediated clustering operates on top of species-specific loop-anchoring systems.

Similarly, condensin-mediated condensation may contribute to mitotic chromosome compaction by organizing chromatin into densely packed, cylindrical structures, as shown in polymer modeling studies [21, 63]. These loops are relatively short, consistent with DNA bridging driven by thermal fluctuations, and suggest that extrusion-independent mechanisms such as BIPS can contribute to chromatin organization, even in non-dividing cells. Notably, mitotic chromosomes exhibit a high degree of axial compaction and stiffness, properties not fully accounted for by loop extrusion alone. Loop extrusion by two condensins can generate Z-loops, but pairwise Z-loop formation alone cannot account for the multi-loop clustering and axial compaction characteristic of mitotic chromosomes [64]; we propose that BIPS provides the multivalent bridging scaffold that assembles condensin–DNA units into these axial structures. Our data support a model in which condensin-driven BIPS assembles clusters that enhance chromatin rigidity and maintain mitotic chromosome integrity during segregation [21].

Cohesin also accumulates at DNA double-strand break sites, where it supports homology-directed repair [17]. Notably, such recruitment can occur independently of ATP hydrolysis, suggesting that bridging and condensation mechanisms may underlie SMC retention at DNA lesions. BIPS could provide a means for concentrating repair factors within microenvironments, stabilizing DNA ends and coordinating the local chromatin context during repair. Cohesin is also frequently enriched at super-enhancers—regulatory domains characterized by dense transcriptional activity and phase-separated transcription factor hubs [33]. BIPS may contribute to the structural organization of these regions by bridging enhancer elements and facilitating condensate formation, thereby promoting enhancer–promoter interactions and transcriptional robustness.

These scenarios illustrate that SMC-mediated condensation is not merely a passive byproduct of DNA binding but a regulated, context-dependent mechanism for organizing chromatin. BIPS expands the functional repertoire of SMC complexes and underscores their versatility in shaping nuclear structure.

The multiple capacity of SMC complexes for other mechanisms and phase condensation raises important questions about how these mechanisms are functionally integrated [65]. Although loop extrusion has been validated as a central driver of long-range chromatin contacts, our findings suggest that BIPS may operate in parallel, or even cooperatively, with extrusion [14]. For instance, extruded loops may increase the local density of DNA, thereby facilitating BIPS nucleation. Conversely, BIPS-mediated clustering of SMCs at genomic loci may stall extrusion. In addition, a topologically loaded SMC could be a nucleation point to trigger further SMC recruitment using the BIPS mechanism. Further support for an interplay comes from studies of transcriptional condensates, where CTCF-anchored loops have been shown to nucleate phase-separated domains enriched for Pol II, Mediator, and other coactivators [33]. Transcription may also generate the targets for loop capture, which in turn nucleate BIPS [66]. Similarly, SMC-driven loops could act as structural templates for broader nuclear condensates, linking architectural and regulatory functions of chromatin [22, 67]. Future studies are necessary to reveal how different molecular mechanisms involving BIPS interplay harmoniously to organize the genome structure.

Our study found BIPS to be a conserved and mechanistically distinct mode of chromatin organization by SMC complexes. By integrating this mechanism with canonical loop extrusion, we propose that SMCs operate through dual complementary pathways to establish genome architecture. BIPS enables passive ATP-independent condensation of chromatin domains, while extrusion dynamically generates and regulates long-range interactions. This duality offers flexibility and robustness in chromatin folding across cell types and biological processes.

Finally, several questions remain. How are BIPS and loop extrusion regulated by chromatin context, epigenetic marks, or nuclear localization? Do other chromatin-associated proteins—such as CTCF, transcription factors, or histone modifiers—similarly utilize DNA bridging to nucleate condensates? What determines the transition between extrusion-dominant and condensation-dominant modes of SMC activity? Addressing these questions will help to fully understand the architectural logic of the eukaryotic genome. The mechanistic principles uncovered here may extend to a broader set of nuclear condensates, highlighting bridging as a general strategy for spatial genome control.

## Supplementary Material

gkag850_Supplemental_Files

## Data Availability

All data are available in the main text or the supplementary materials.

## Notes

Present address: Chugai Life Science Park Yokohama, Chugai Pharmaceutical Co., Yokohama 221-0056, Japan
