# Supplementary material for: Bridging-driven condensation by eukaryotic SMC complexes is a conserved feature of genome organization
Source: Nucleic Acids Res. 2026 Sep 3;54(16):gkag850. doi: 10.1093/nar/gkag850 (PMC13539107; doi:10.1093/nar/gkag850)
Supplement: Supplementary file 1 [file gkag850_supplemental_files.zip › BIPS_NAR_SM_V02.docx]

Supplementary Figures S1–10

Supplementary Table S1

Supplementary Movies S1–S8

Supplementary Figure S1. DNA compaction by *Sc* condensin, *Sp* condensin, *Sp* cohesin*,* and *Hs* cohesin complexes. (A–C) Snapshots before and after adding subunit-missing controls: before and after *Sc* condensin without Ycg1 (A), *Sp* cohesin without Mis4–Ssl3 (B), and *Hs* cohesin without NIPBL–MAU2 (C) (*N* = 20, 20, and 20 for panels A–C). (D, F, H, J) Snapshots before and after SMC/DNA cluster formation with labeled SMC complexes, showing colocalization of compacted DNA (blue) and SMCs (magenta). In the schematics on the right, blue, yellow, and red represent DNA, biotin-streptavidin, and the SMC complex. (E, G, I, K) Kymographs of DNA SMC channels during compaction (*N* = 27, 112, 54, and 59 for panels E–H).

Supplementary Figure S2. Number of SMC complexes per cluster.

(A–C) Representative traces for single-molecule bleaching traces of Atto647N-labeled *Sc* condensin complex (A), Alexa647-labeled *Sp* condensin (B) and JF646-labeled *Hs* cohesin complex (C). (D–F) Distributions of photobleaching steps of Atto647N-labelled *Sc* condensin (D; mean ± SD = 0.2 ± 0.1, *N* = 168), Alexa647-labelled *Sp* condensin (E; mean ± SD = 0.61 ± 0.44, *N* = 273), and JF646-labelled *Hs* cohesin complexes (F; mean ± SD = 0.26 ± 0.13, *N* = 457). Solid lines represent Gaussian fits. (G–R) Number of SMC complexes estimated by comparison between SMC cluster intensities divided by a single bleaching-step intensities (mean ± SD = 1080 ± 860, 254 ± 106, 777 ± 941, and 405 ± 316; *N* = 32, 88, 274, and 202 for *Sc* condensing (G), *Sp* condensing (H), *Sp* cohesion (I)*,* and *Hs* cohesin (J) complexes, respectively). Solid lines are gaussian fits, and the mean values were obtained by Gaussian fitting.

Supplementary Figure S3. Salt concentration–dependent SMC/DNA clustering.

**(A,B)** Two-dimensional phase diagram of DNA cluster formation by *Sp* condensin (A) without and with (B) 5% PEG (35 kDa). A higher concentration of the molecular crowder PEG may stimulate cluster formation (**C–F)** Kymographs of DNA (top) and SMC clusters (bottom) during 500 mM NaCl washing (*N* = 6, 24, 7, and 8 molecules for *Sc* condensin, *Sp* condensin, *Sp* cohesin*,* and *Hs* cohesin complexes, respectively). (**G,H)** Recovery experiment after 500-mM NaCl washing of *Hs* cohesin/DNA cluster. (G) Snapshots before and after recovery experiment of *Hs*-cohesin complex clustering and (H) kymographs of recovery of *Hs*-cohesin complexes upon reintroduction of Hs cohesin after 500 mM NaCl washing. (**I)** Average time traces of recovery of 4 different SMC complexes after 500 mM NaCl washing experiment (mean ± SEM). Solid lines indicate cumulative single exponential fits (*τ* = mean ± SD = 38 ± 1 s, 172 ± 8.7 s, 150 ± 9 s, and 118 ± 6 s for *Sc* condensin (*N* = 32), *Sp* condensin (*N* = 44), *Sp* cohesin (*N* = 49), and *Hs* cohesin (*N* = 30), respectively).

Supplementary Figure S4. Hexanediol treatment. (A) Snapshots before and after of 1,6 hexanediol treatment of Atto647N labeled *Sc* condensin cluster. (B–E) Representative kymographs of 1,6 hexanediol treatment. After hexanediol treatment, dissociation of SMC complexes was observed. (F) Average time trace of dissociation of SMC complexes after 1,6 hexanediol treatment (mean ± SEM). Solid lines represent single-exponential fits (*τ* = mean ± SD = 3.0 ± 0.1 s, 8.3 ± 0.5 s, 3.3 ± 0.3 s, and 6.7 ± 0.3 s for *Sc* condensin (*N* = 11), *Sp* condensin (*N* = 33), *Sp* cohesin (*N* = 37), and *Hs* cohesin (*N* = 58)).

Supplementary Figure S5. Properties of phase condensation.

**(A–D)** Kymographs showing merging of two distinct SMC clusters (*N* = 158 (A), 22 (B), 65 (C), and 75 (D) merging events). White circles show merging points. **(E, F)** Snapshots of more merging events of *Sc* condensin/DNA clusters (E) and *Sp* condensin/DNA clusters (F). **(G, H)** Kymographs showing exchange of labeled *Sc* condensin/DNA (G; *N* = 57) and *Sp* condensin/DNA clusters (H; *N* = 10) with unlabeled *Sc* and *Sp* condensin without change in DNA clusters.

Supplementary Figure S6. AFM images of *Hs* cohesin complex.

**(A)** Representative AFM images of *Hs* cohesin complex. (**B)** Volume distributions of *Hs* cohesin complex (mean ± SD = 970 ± 310 nm^3^, *N* = 855). The solid blue line represents a Gaussian fit.

Supplementary Figure S7. DNA length–dependent *Hs* cohesin complex–DNA cluster size. (A–E) Representative images of *Hs* cohesin complex with (A) 300 bp, (B) 1 kbp, (C) 7 kbp, (D) 10 kbp, and (E) 50 kbp. (F, G) Representative AFM images of DNA-bridged loops formed by *Sc* condensin (F) and *Sp* cohesin (G) (*n* = 24 and 41, respectively).

**Supplementary Figure S8. BIPS through inter-DNA interactions by *Hs* cohesin complex.**

**(A, B)** Representative images of 1 nM *Hs* cohesin complex with 30 ng/μL 1-kbp DNA (A), and 10 nM *Hs* cohesin complex with 30 ng/μL (B) 1-kbp DNA. **(C)** Cumulative probability plot via cluster radius, $R_{C}$. *n* = 38 (1 nM *Hs* cohesin only), 33 (1 nM *Hs* cohesin + 3 ng/μL DNA), 36 (1 nM *Hs* cohesin + 30 ng/μL DNA), and 43 (10 nM *Hs* cohesin + 30 ng/μL DNA)**. (D)** Large clusters were defined as those with $R_{C}>83$ nm, a threshold above the maximum cluster size observed in the cohesin-only condition (*n* = 0, 2, 0, and 6, respectively). Error bars represent counting error. **(E)** Single-molecule images of inter-DNA bridging and zippering by 1 nM *Hs* cohesin. Once a *Hs*-cohesin complex bridge two distinct, closely positioned, parallel DNA molecules on an SMF microscope, zippering by additional *Hs*-cohesin complexes follows immediately, proceeding from the bridged point toward the ends of the DNA molecules. **(F)** Schematic model of BIPS driven by inter-DNA bridging.

Supplementary Figure S9. Super-resolution images of *Hs* cohesin complex condensates in HCT116 cells.

Supplementary Figure S10. Micro-C contact map and RAD21 ChIP-seq data from *H. sapiens*. (A) Representative Hi-C contact map (10 kb resolution, HFFc6 cells) aligned with RAD21 ChIP–seq peaks (GM12878 cells) spanning 11.5–13 Mb region of *Hs* chromosome X. Numbers indicate cohesin-associated regions (CARs) corresponding to ChIP–seq peaks. TADs are defined by merging CARs connected by loops. TAD boundaries (solid black lines) were defined as TADs of locally maximal size encompassing multiple loops (circles), with dashed lines indicating the outermost loop anchors of each TAD. A long-range loop between loci 2 and 8 encompasses nested loops. (B) Distribution of maximum cohesin-organized loop size per a TAD. (C) Distribution of the number of cohesin-organized loops per TAD in HFFc6 cells. The resolution of the contact map is 10 kbp. (D) Chromosome X: 9–20 Mbp, 10–14 Mbp, 11.5–13 Mbp (zoomed view). (E) Chromosome 2: 25–37 Mbp, 25–30 Mbp, 27–29.5 Mbp (zoomed view). (F) Chromosome 12: 25–35 Mbp, 25–30 Mbp, 26.5–28 Mbp (zoomed view).

| Species | Complex | Total count | Estimated nuclear concentration (nM) | Method | Reference |
| --- | --- | --- | --- | --- | --- |
| *Sc* | cohesin | 2,132 | 1039 | Integrated estimate | [46] |
|  | condensin | 1,411 | 688 | Integrated estimate | [46] |
| *Sp* | cohesin | 811 | 183 | LC-MS | [45] |
|  | condensin | 3,047 | 633 | LC-MS | [45] |
| *Hs* | cohesin | 261,445 | 332 | FCS | [42] |

**Supplementary Table 1. SMC abundance and nuclear concentration.** The table summarizes the total copy numbers of cohesin and condensin in *Sc*, *Sp*, and *Hs*, quantified from the kleisin subunit of each complex. Abundances were determined by an integrated proteomic estimate (*Sc*), liquid chromatography–mass spectrometry (LC–MS; *Sp*), and fluorescence correlation spectroscopy (FCS; *Hs*); nuclear concentrations were then calculated by dividing these counts by the nuclear volume of each species. For *Sc*, the integrated estimate unifies mass spectrometry, GFP microscopy, GFP flow cytometry, and TAP immunoblotting datasets through a statistical reconciliation procedure [46].

**Supplementary Movies Legends**

**Movie S1.** DNA cluster formation by *Sp* condensin complex.

**Movie S2.** Cluster formation by *Sc* condensin complex on side-flow double-tethered DNA.

**Movie S3.** Two-channel cluster formation by *Hs* cohesin complex. Shown are the overlay (left), DNA (green, middle), and protein (red, right) channels.

**Movie S4.** High-salt wash experiment for *Sp* cohesin complex.

**Movie S5. Recovery of DNA cluster formation by *Hs* cohesin complex.** Shown are the overlay (left), DNA (green, middle), and protein (red, right) channels.

**Movie S6. 1,6-Hexanediol treatment for *Sp* cohesin complex.**

**Movie S7. Merging of (A) *Sp* cohesin complex and (B) *Hs* cohesin complex.** Shown are the overlay (left), DNA (green, middle), and protein (red, right) channels.

**Movie S8. Exchange assay with (A) *Sc* condensin complex and (B) *Sp* condensin complex.** Shown are the overlay (left), DNA (green, middle), and protein (red, right) channels.
